# An experimental test of the EICA hypothesis in multiple ranges: invasive populations outperform those from the native range independent of insect herbivore suppression

**DOI:** 10.1093/aobpla/plw087

**Published:** 2016-12-30

**Authors:** Evan Siemann, Saara J. DeWalt, Jianwen Zou, William E. Rogers

**Affiliations:** 1 Department of Biosciences, Rice University, Houston, TX 77005, USA; 2 Department of Biological Sciences, Clemson University, Clemson, SC 29634, USA; 3 College of Resources and Environmental Sciences, Nanjing Agricultural University, Nanjing, Jiangsu 210095, China; 4 Department of Ecosystem Science and Management, Texas A&M University, College Station, TX 77843, USA

**Keywords:** Biogeography, EICA, evolutionary dynamics, insect herbivores, plant invasions, *Triadica sebifera*

## Abstract

The success of invasive plants may reflect environmental differences in their native and introduced ranges including both abiotic and biotic conditions, such as release from aboveground herbivory. However, in response to these novel conditions, plants from invasive populations may have higher growth rates and lower defence levels compared with those in the native range. This may contribute to their success in the introduced range but perhaps not in the native range. Here, we grew 1000 *Triadica sebifera* plants from 14 native and introduced populations in seven common gardens with unmanaged background vegetation for three growing seasons in three geographic venues that varied in *T. sebifera* status and insect herbivore communities: Texas—*T. sebifera* is invasive, low levels of generalist herbivory; Hawaii—*T. sebifera* introduced but not invasive, high levels of generalist herbivory from exotic herbivores; China—native range, both generalist and specialist herbivores. We suppressed aboveground insects with insecticide on half the plants. Aboveground damage in the first growing season was lowest in Texas and insecticide sprays reduced damage in China. At the end of the first growing season, plants were tallest on an average in China and shortest in Hawaii. However, height in later years and mass were the highest on average in Texas and the lowest in Hawaii. However, there was large variation in damage and plant performance among gardens within venues. Our results suggest that more rapid aboveground growth rates contribute to *T. sebifera*’s success in both the invasive and native ranges independent of aboveground herbivory. However, strong variation among sites indicates that *T. sebifera* plants from invasive populations only have a strong advantage in a subset of sites in Texas.

## Teaser

This paper reports the results of a multi-year manipulative common garden experiment that investigates the roles of variation in plant defense and growth in the competitive ability of an invasive tree in the native range and two introduced ranges. We found that plants from invasive populations had more rapid aboveground growth rates that contributed to their success in both introduced and native ranges independent of aboveground herbivory. However, strong variation among sites indicated that plants from invasive populations only have a strong advantage in a subset of sites in the introduced range.

## Introduction

Two key factors are widely believed to increase the abundance and vigor of many invasive plants in their introduced range compared with their native range. First, some species may be innately better competitors because they evolved in a more competitive environment (Darwin 1859; Crawley 1987; Vitousek and Walker 1989; Lodge 1993). Once established in their introduced range, invasive plants may gain a systematic advantage over competitively inferior native plants. Second, invasive plants typically experience low losses to herbivores in their introduced range (Elton 1958; Maron and Vila 2001; Keane and Crawley 2002; Liu and Stilling 2006). With low levels of damage in the introduced range, resources normally lost to enemies or the production of induced defences may be allocated to growth and/or reproduction by a plastic phenotypic response (‘Enemies Hypothesis’; Alpert *et al.* 2000; Stowe *et al.* 2000). Early theoretical and empirical research in invasion ecology primarily focused on these two hypotheses.

An additional hypothesis within this framework (Evolution of Increased Competitive Ability ‘EICA’) proposed that invasive plants evolve reduced allocation to defence and increased allocation to growth and/or reproduction because they are seldom attacked by enemies (Blossey and Nötzold 1995). Because allocation to defence may be as costly as herbivore damage (Bazzaz *et al.* 1987; Simms 1992; Baldwin 1998), plants that escape their enemies in an introduced range would gain a selective benefit from decreasing their defensive investment. The EICA Hypothesis predicts that plants from populations in the introduced range (‘invasive populations’) will grow faster and/or produce more seeds but be less well-defended against enemies than those from populations in its native range (‘native populations’). This hypothesis has since been expanded to include a number of additional dimensions so that it considers a number of conditions and evolutionary predictions (Orians and Ward 2010). A key prediction is that in a common garden where herbivores from the plant’s native range are absent, plants from invasive populations should be superior, whereas, plants from native populations should outperform those from invasive populations in habitats where herbivores from the plant’s native range are abundant. Greenhouse experiments, studies with plants in common gardens in a single range, studies with plants in common gardens in both ranges and studies that manipulate herbivores in a single range have provided mixed evidence for the evolution of decreased herbivore resistance and greater competitive ability in invasive plants, for reviews see Bossdorf *et al.* (2005), Orians and Ward (2010), Wheeler and Schaffner (2013), Bock *et al.* (2015), and Colautti and Lau (2015). An important next step is to conduct experiments in replicated common gardens in multiple ranges with plants from invasive and native populations together with experimental manipulation of herbivores (Orians and Ward 2010). Here, we conducted such an experiment with Chinese tallow tree (*Triadica sebifera—*formerly *Sapium sebiferum*, Euphorbiaceae), which is a major invader in grasslands, wetlands and forests in the southeastern United States (Bruce *et al.* 1997).

A number of studies have shown that compared with *T. sebifera* plants from populations in the native range differ from plants from populations in the introduced range in a number of traits that may affect their performance in field conditions. Specifically, relative to those from native populations, those from invasive populations have lower concentrations of phenolics in their foliage and roots (Wang *et al.* 2012; Huang *et al.* 2014), produce lower amounts of extrafloral nectar (Carrillo *et al.* 2012), suffer higher levels of herbivore attack in greenhouse and field cage studies (Siemann and Rogers 2003*c*; Huang *et al.* 2010; Wang *et al.* 2011; Huang *et al.* 2012*a*), have higher tolerance to herbivory from artificial defoliation (Rogers and Siemann 2004), generalist herbivores (Zou *et al.* 2008; Carrillo *et al.* 2014), and specialist herbivores (Huang *et al.* 2010; Wang *et al.* 2011) and have more rapid growth rates (Rogers and Siemann 2005; Zhang *et al.* 2013). Together, these results suggest that *T. sebifera* plants from invasive populations should suffer more herbivore damage in common gardens and outperform those from native populations in gardens or experimental treatments in which herbivore pressure is lower.

The performance of *T. sebifera* has been investigated in the native range and two introduced ranges, the continental US and Hawaii. In the southeastern continental US, *T. sebifera* plants suffer low levels of herbivore attack from generalist folivores. There, *T. sebifera* is highly invasive, and plants from invasive populations outperformed those from native populations in a 17-year common garden experiment in which the background vegetation was managed (Siemann and Rogers 2003*b*). In Hawaii, *T. sebifera* plants suffer high levels of herbivore attack from exotic generalist folivores, *T. sebifera* is a casual alien as defined by (Richardson *et al.* 2000*b*), and plants from native populations outperformed those from invasive populations in a 17-year common garden experiment in which the background vegetation was managed (Siemann and Rogers 2003*b*). The first record of *T. sebifera* in Hawaii dates to the 1920s (Bishop Museum specimen BISH 50417 collected in 1927, North Kohala, Hawaii) but fewer than 100 trees are documented in the islands prior to 1980. The failure of *Triadica* to spread in Hawaii almost a century after introduction is interesting, since it occupied more than 15% of the land area of some counties in Texas within a similar period of time (Bruce *et al.* 1995). In China, *T. sebifera* plants suffer attack from specialists and generalist herbivores from a broad variety of feeding guilds (Zhang *et al.* 2015) and generalist and specialist herbivores, aboveground and belowground, prefer to feed on *T. sebifera* from invasive populations in caged feeding trials (Huang *et al.* 2012*a*, Huang *et al.* 2010, Wang *et al.* 2011).

Here we addressed the following question: How does *Triadica sebifera*’s competitive ability depend on population origin and aboveground herbivorous insect attack in these three geographic venues? We predicted that (1) *T. sebifera* plants would receive less damage and perform better in Texas (non-native range) than in Hawaii (non-native range) or China (native range) without insect suppression, (2) *T. sebifera* plants from invasive populations would perform better relative to those from native populations in Texas without insecticide sprays but the opposite pattern would occur in China, (3) insect herbivore suppression would increase performance of *T. sebifera* plants from invasive populations more than that of plants from native populations in Hawaii and China.

## Methods

### Seed collection

We hand collected *T. sebifera* tree seeds in December 2003 and January 2004 from populations across the invasive (southeastern continental US—five populations) and native (China—nine populations) ranges (Table 1). Genetic analyses indicate that the original introduction into the continental US in Savannah, GA in 1772 was from a different source population (southern China—Guangzhou population here is a close match) than the later introduction into the Gulf Coast (∼1900) which were from eastern China (Nanjing population here is a close match) in the northeastern part of *T. sebifera*’s native range (DeWalt *et al.* 2011). We collected seeds from populations in the introduced range from populations descended from each introduction (three from original, two from later) and from native populations that span the range of *T. sebifera*. Although *T. sebifera* was introduced to Hawaii by the early 20th century, there were no self-sustaining populations at the time of this study that could have served as a source of seeds.

**Table 1.** Populations of *Triadica sebifera* trees used for these experiments. The populations from Georgia are descendants of the original population introduced to Savannah, GA, in 1772, most likely from populations in the southwestern part of tallow’s native range in China (Guangdong is most likely match here). the populations from Texas (and all other populations on the gulf coast) are the result of a subsequent introduction in approximately 1900 using seeds from Jiangsu province (DeWalt *et al*. 2011).

Range	Location	Coordinates	N
Invasive (USA)	Hutchinson Island, GA	N32.10 W81.10	149
	Houston, TX	N29.71 W95.40	261
	Orange, TX	N30.10 W93.74	72
	Savannah, GA	N31.96 W81.07	9
	Sapelo Island, GA	N31.40 W81.28	49
Native (China)	Guangzhou, Guangdong	N23.13 E113.26	50
	Ganzhou, Jiangxi	N25.83 E114.93	23
	Hefei, Anhui	N31.87 E117.29	75
	Zhangjiajie City, Hunan	N29.11 E110.48	21
	Hangzhou, Zhejiang	N30.27 E120.16	63
	Nanchang, Jiangxi	N28.69 E115.87	26
	Nanjing, Jiangsu	N32.03 E118.84	80
	Taihe, Anhui	N26.65 E114.64	43
	Xiamen, Fujian	N24.48 E118.10	79

### Seed planting

We individually planted seeds in Nanjing, Jiangsu China (December 2003), Houston, TX, USA (January 2004) and Honomu, HI, USA (January 2004) into 115 mL containers (Stuewe & Sons, Tangent, OR) filled with locally available topsoil. We germinated seeds in a three-sided greenhouse with screening on the other side (China), an unheated greenhouse with open flaps (Texas), or a paved area that was next to a building and surrounded by screening (Hawaii). We watered containers daily if they were dry.

### Gardens

The two garden locations in China were the Sun Yat Sen Arboretum and the Jiangsu Forestry Institute (Table 2). The former had been a grassy area mowed multiple times per year before the garden was set up. The latter was a 1-year fallow rice field. The two garden locations in Hawaii were the Hawaii Agricultural Research Corporation in Maunawili (on Oahu) and the Malamaki Agricultural Experiment Station (on Hawaii). The former was a 1-year fallow sugar cane field and the latter was an exotic grass dominated fallow field previously used for growing tropical fruit (more than 5 years before). The three gardens in Texas were at the University of Houston Coastal Center (La Marque, TX), Katy Prairie Conservancy (Katy, TX) and Armand Bayou Nature Center (Pasadena, TX). Each was native dominated grassland that had been mowed annually. The background vegetation was not managed in any garden during the experiment so *T. sebifera* plants competed with the background vegetation.

**Table 2.** Research garden sites.

Site	Location	Lat/Long	Annual precip.	# of seedlings
*China*				
Jiangsu Forestry Inst.	Molingguan, Jiangsu	N31.8526 E118.7733	100 cm	200
Sun Yat Sen Garden	Nanjing, Jiangsu	N32.0603 E118.8272	106 cm	120
*Hawaii*				
HARC	Maunawili, Hawaii	N21.3728 W157.7706	190 cm	120
Hawaii Ag Expt Station	Malamaki, HI	N19.4697 W154.8843	203cm	120
*Texas*				
Katy Prairie Conservancy	Katy, TX	N29.9267 W95.9239	125 cm	120
Armand Bayou Nature Center	Pasadena, TX	N29.5936 W95.0526	137 cm	120
University of Houston Coastal Center	La Marque, TX	N29.3773 W95.0401	111 cm	200

### Experiment design

The experiment was a factorial design with three geographic venues (Hawaii, Texas, China), seven gardens nested within venue, two population origins (continental US vs. China), 14 populations nested in origin and an insect suppression treatment. In each of the seven gardens we planted seedlings from every invasive and native population in March or April 2004. The design was balanced within each venue and garden for continental origin, spray, and their interaction. However, the design was not balanced for populations or number of seedlings per garden. This reflected constraints on available seedlings and space. In total, there were 1000 plants. We planted seedlings on one meter grid spacing in randomized locations. We did not water plants. The experiment continued for three growing seasons with harvest approximately 900 days later in October 2007.

### Insect suppression

We sprayed plants in the insect suppression treatment approximately every 2 weeks during the growing seasons with esfenvalerate, a broad spectrum pyrethroid, to suppress aboveground insects (DuPont-Corp 1989). This product has been widely used in ecological research (Cain *et al.* 1991; Carson and Root 2000) including with *T. sebifera* seedlings (Siemann and Rogers 2003*a*). This product was available in similar formulations in Texas, Hawaii and China. The amount of active ingredient per volume in the commercially available concentrate varied between the US and China but the concentrations of active ingredient in the insecticide sprays we applied were identical at every location (38 mg/L). We sprayed control plants with water.

### Data collection

We recorded seedling survival several times during each growing season. We also counted the number of leaves and visually surveyed seedlings for amount of foliar chewing insect damage as the average amount of damage per leaf multiple times each growing season. At the beginning of the experiment and at the end of each of the three growing seasons, we measured plant height. At the end of the experiment, we clipped plants at ground level, separated stems and leaves, dried them and weighed them. All appropriate permits were obtained and plants were harvested before flowering to prevent introduction of new genetic material.

### Analyses

We conducted two types of tests of significance that differed in null hypotheses for genetic effects. In the first, we tested whether any predictor variables were significant predictors of variation in the response variable relative to residual error. In these analyses, the null hypothesis for population continental origin was that plants of different population origins did not differ (or that plants of different origins did not differ in interaction with other factors). Then, if continental origin was significant in the initial analyses, we performed an additional test of significance that examined whether the variation explained by continental origin was significantly larger than that explained by source population nested in continental origin (as a random effect). The first set of analyses focused on whether there were differences in ecological interactions due to plant continental origin. The second set of analyses focused on whether these genetic differences between population origins were consistent with repeated evolution of the same traits in the introduced range.

We initially fit a model with all possible terms and all nested models (with the constraint that models did not contain interaction terms without the corresponding lower level factors). We compared AIC values of these models and selected the model with the lowest AIC for which all significant terms in the full model were significant. For repeated variables (height and damage), we chose the lowest AIC model that met this criterion for all 3 years of data.

These ANOVA models examined whether variation in damage (square root transformed), height (square root transformed), mass (square root transformed) and percent mass that was leaves (arc sine transformed) depended on factors that corresponded to different distinct variable types in terms of mechanism. *Geographic* predictors included venue [‘V’: China, Hawaii, Texas] and garden nested in venue (‘G(V)’). *Genetic* predictors included population origin (‘O’: China or continental US). The *biotic* predictor was insect suppression with insecticide sprays (‘S’). We also included terms for the *interactions* of geographic and biotic factors (e.g. venue × spray), genetic and biotic factors (origin × spray), genetic and geographic factors (e.g. origin × venue) and the interaction of all three types (e.g. origin × venue × spray). Damage data were square root transformed. Damage and height analyses were unbalanced at the beginning of the experiment (e.g. damage in the first year) because the design was not balanced among gardens and populations (see above) and analyses became increasingly unbalanced in the later years of the experiment because mortality was not independent of predictors. Therefore, we examined each year of the experiment as a separate analysis using Proc Mixed (SAS 9.4) with restricted maximum likelihood model fitting. For survival we used analyses based on Cox proportional hazards (Proc Surveyphreg) with the Taylor series method and Type 3 tests of factor significance to examine variation in time of mortality.

## Results

**Figure 1:**
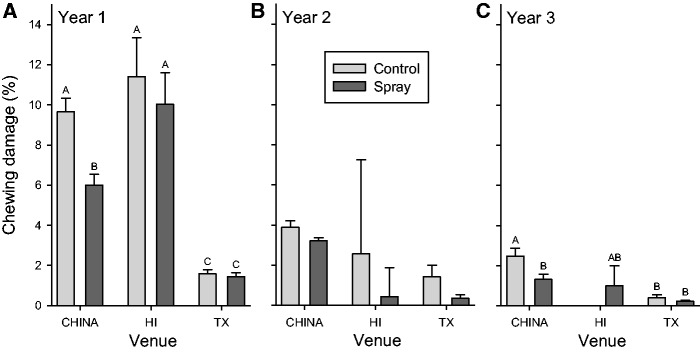
The dependence of chewing damage in each of the three growing seasons on geographic venue (Hawaii, Texas, China) and insecticide spray (control vs. spray) and their interactions. Unadjusted means + 1 SE.

Insect damage varied with the interaction of geographic and biotic factors (Table 3 and Fig. 1; model: V G(V) S V × S G × S(V), [**see Supporting Information—Table S1**]). In the first year, insect chewing damage was the highest on an average in Hawaii, intermediate in China, and the lowest in Texas and damage varied between Hawaii gardens (Malamaki [20.9 ± 2.1 %] > Maunawili [0.5 ± 0.2 %]). We observed high densities of the Caribbean leatherleaf slug (*Sarasinula plebeian*, Veronicellidae*—*native to Latin America, accidentally introduced to Hawaii in 1978) feeding on seedlings in the Malamaki garden. Damage was higher in China than the other two venues in the third year on average. Insecticide sprays significantly reduced damage in China in the first and third growing seasons (effect sizes 1.54 and 1.88) as predicted.

**Table 3.** The dependence of chewing damage in each growing season of the experiment on geographic venue (Texas, Hawaii, China), garden nested in venue, spray (control or insecticide), and their interactions using residual error for *F*-tests. Significant results are indicated in bold. Results are shown in Figure 1.

	Year 1			Year 2			Year 3			
Term	*Df*	*F*	*P*	*Df*	*F*	*P*	*df*	*F*	*P*
*Geographic*									
Venue	2,986	**117.2**	**<0.0001**	2,453	0.9	0.3908	2,340	**5.9**	**0.0030**
Garden(venue)	4,986	**86.4**	**<0.0001**	4,453	1.7	0.1509	3,340	**2.8**	**0.0379**
*Biotic*									
Spray	1,986	**7.3**	**0.0072**	1,453	0.5	0.5024	1,340	**9.9**	**0.0018**
*Geographic × biotic*									
Venue × spray	2,986	**7.1**	**0.0009**	2,453	0.3	0.7273	1,340	**6.1**	**0.0142**
Garden × spray (venue)	4,986	0.5	0.7164	3,453	0.7	0.5677	1,340	0.8	0.3882

**Figure 2:**
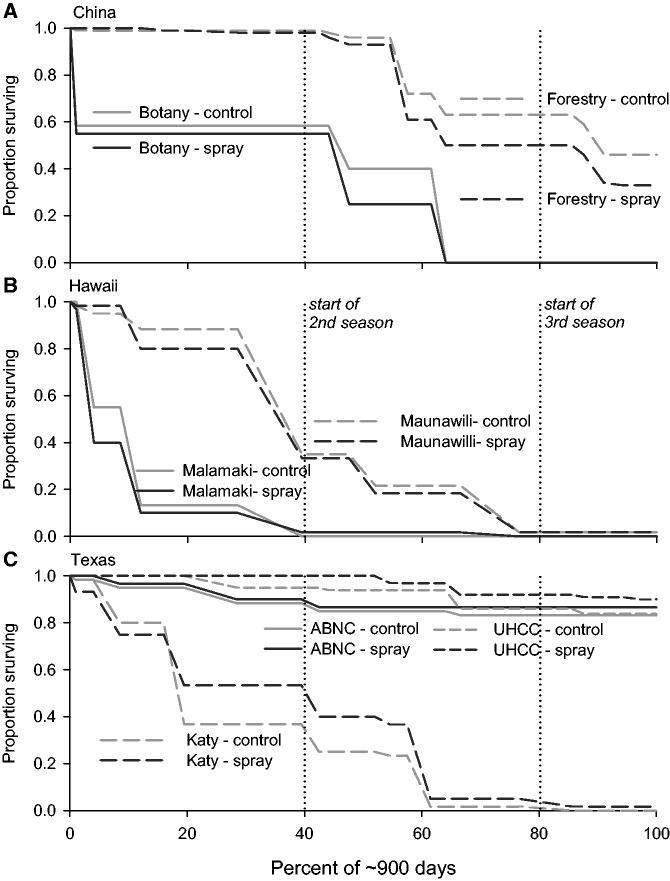
The dependence of plant survival within gardens on insecticide spray (control vs. spray) in different geographic venues (A. China, B. Hawaii, C. Texas).

Plant survival varied with the interaction of geographic and biotic factors (model: V G(V) S V × S, [**see Supporting Information—Table S1**]). Plants survived the longest in Texas (UHCC > ABNC > Katy), an intermediate time in China (Forestry > Botany), and the shortest time in Hawaii (Maunawili > Malamaki) with significant variation among gardens within venues (venue: *F*_2,999 _ = _ _155.5, *P* < 0.0001; garden(venue): *F*_4,999 _ = _ _93.5, *P* < 0.0001, Fig. 2). There was no overall effect of insecticide sprays (*F*_1,999 _ = _ _0.5, *P*  =  0.4999). But, there was a significant interaction between venue and spray (*F*_2,999 _ = _ _5.3, *P*  =  0.0053) reflecting shorter survival time in China with insecticide sprays (514 vs. 469 days), no effect of insecticide sprays on survival in Hawaii (238 vs. 218 days) and a positive effect of insecticide sprays in Texas (626 vs. 665 days).

**Figure 3:**
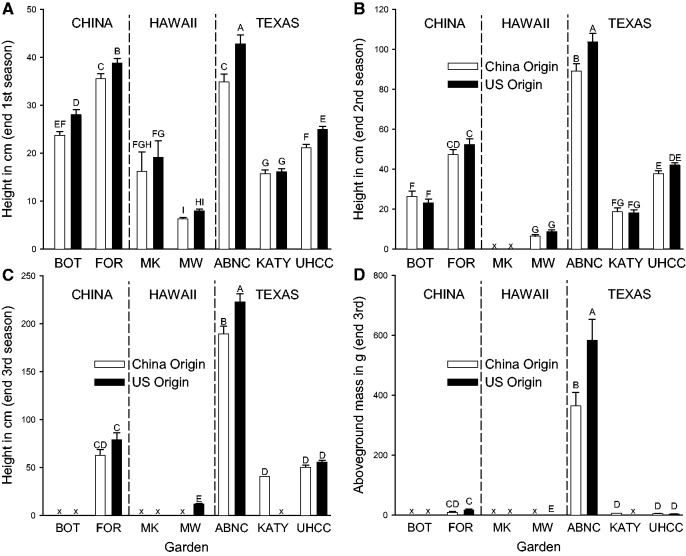
The dependence of height at the end of each of the three growing seasons (A–C) and mass at the end of the third growing season (D) in gardens within different geographic venues (Hawaii, Texas, China) on population origin (China vs. continental US). BOT = Botany, FOR = forestry, MW = Maunawili, MK = Malamaki. Unadjusted means + 1 SE.

Plant height depended on biotic, and the interaction of genetic and geographic factors (Table 4 and Fig. 3; model V G(V) O S V × S G × S(V) V × S O × S O × V G × O(V), [**see Supporting Information—Table S1**]). At the end of the first growing season, plants were the tallest on an average in China (Forestry > Botany), intermediate height in Texas (ABNC > UHCC > Katy) and the shortest in Hawaii (Malamaki > Maunawili) with US origin plants taller than China origin plants in both China gardens and two of the three Texas gardens (Fig. 3A). Plants sprayed with insecticide were taller at the end of the first growing season (245.7 ± 7.3 mm) than plants sprayed with only water (219.7 ± 6.2 mm). At the end of the second and third growing seasons, plants were the tallest on average in Texas (2nd: ABNC > UHCC > Katy; 3rd: ABNC > UHCC∼Katy), intermediate height in China (2nd: Forestry > Botany; 3rd: only Forestry surviving), and the shortest in Hawaii (only Maunawili surviving). US origin plants were taller than those of China origin on an average at the end of the first and third growing seasons and these differences were significantly larger than the variation among populations. US origin plants were significantly taller than China origin plants in the ABNC garden in the second and third years (Fig. 3B,C).

**Table 4.** The dependence of plant height in each growing season of the experiment on venue (Texas, Hawaii, China), garden nested in venue, population origin (continental USA or china), spray (control or insecticide), and interactions using residual error for *F*-tests. For the significant effects of origin, an additional *F*-test used the amount of variation among populations to test for differences between continental origins. Significant results are indicated in bold. Results are shown in Figure 3.

	Year 1			Year 2			Year 3		
Term	D*f*	*F*	*P*	D*f*	*F*	*P*	D*f*	*F*	*P*
*Geographic*									
Venue	2,777	**103.5**	**<0.0001**	2,497	**29.8**	**<0.0001**	2,330	**10.5**	**<0.0001**
Garden(venue)	4,777	**145.9**	**<0.0001**	4,497	**204.0**	**<0.0001**	3,330	**497.3**	**<0.0001**
*Genetic*									
Origin	1,777	**12.7**	**0.0004**	1,497	1.6	0.2057	1,330	**11.9**	**0.0006**
*[Origin vs. population]*	*1,12*	***5.0***	***0.0421***				1,12	***11.8***	***0.0049***
*Biotic*									
Spray	1,777	**7.8**	**0.0052**	1,497	0.7	0.4015	1,330	1.68	0.1961
*Geographic × biotic*									
Venue × spray	2,777	2.7	0.0655	2,497	0.1	0.8725	1,330	1.1	0.3025
Garden × spray (venue)	4,777	2.3	0.0534	4,497	1.1	0.3577	1,330	0.4	0.5429
*Genetic × biotic*									
Origin × spray	1,777	0.5	0.4903	1.497	0.4	0.5101	1,330	0.4	0.5105
*Genetic × geographic*									
Origin × venue	2,777	0.2	0.8218	2,497	0.7	0.4833	1,330	<0.1	0.8766
Origin × garden (venue)	4,777	**2.9**	**0.0230**	3,497	**2.7**	**0.0458**	1,330	**10.5**	**0.0013**

Plant mass depended on the interaction of geographic and genetic factors (Fig. 3D; model: V G(V) O O × V G × O(V), [**see Supporting Information—Table S1**]). Plants had the greatest mass on an average in Texas, intermediate mass in China and the smallest mass in Hawaii (venue: *F*_2,347 _ = _ _3.8, *P*  =  0.0237) with significant variation among Texas gardens (garden(venue): *F*_2,347 _ = _ _129.1, *P* < 0.0001). US origin plants were larger on an average than China origin plants (origin: *F*_1,347 _ = _ _5.1, *P*  =  0.0246) and this variation was significantly larger than the variation among populations (*F*_1,12 _ = _ _5.1, *P*  =  0.0237) but this origin differences were only significant in the ABNC garden (garden × origin(venue): *F*_1,333 _ = _ _14.1, *P*  =  0.0002; Fig. 3D).

Plant allocation to leaves depended on geographic and biotic factors (model: V G(V) O S O × V G × O(V), [**see Supporting Information—Table S1**]). The proportion of aboveground mass that was leaves depended on venue (*F*_2,337 _ = _ _6.3, *P*  =  0.0020) with it highest in Texas (0.228 ± 0.005), intermediate in China (0.204 ± 0.008) and the lowest in Hawaii (0.075 ± 0.075). It also depended on insecticide sprays (*F*_1,337 _ = _ _14.4, *P*  =  0.0002) with plants that were sprayed having lower proportion of biomass as leaves (0.207 ± 0.006) than those that were not sprayed (0.236 ± 0.006). The other factors were not significant (G(V): *F*_2,337 _ = _ _1.1, *P*  =  0.3281; O: *F*_1,337 _ = _ _3.2, *P*  =  0.0733; O × V: *F*_1,337 _ = _ _0.1, *P*  =  0.8058; G × O(V): *F*_1,337 _ = _ _2.6, *P*  =  0.1085).

## Discussion

We did not observe genotype by environment interactions as we expected even in combination with aboveground insect herbivore suppression but rather plants from invasive populations always outperformed those from native populations when there were differences. This is similar to tests of the performance of *Microstegium vimineum* in common gardens in both the native and introduced ranges in which plants from invasive populations always outperformed those from native populations (Flory *et al.* 2011) and invasive genotypes of *Ageratina adenophora* outperforming native ones in the native range independent of insect suppression (Zheng *et al.* 2015*a*). This type of pattern in which invasive populations outperform native ones even in the native range suggests that testing the EICA hypothesis is complex and may not be valid in this case. On the one hand, it might indicate that the basic EICA assumption that plants are adapted to the conditions in the native range and then become adapted to the conditions in the new range is incorrect. However, it might instead indicate that the temporal and spatial scales at which selection operates are not the same as the temporal and spatial scales of ecological tests of performances of plants from different ranges. For instance, the greater height and mass of *T. sebifera* plants from the introduced and native ranges in Texas we observed here is consistent with earlier results from a long-term common garden experiment (Siemann and Rogers 2003*b*) and short-term field and greenhouse studies (Rogers and Siemann 2005; Zou *et al.* 2008; Huang *et al.* 2012*a*; Zhang *et al.* 2013). But, the extremely high mortality in Hawaii gardens we observed here suggests that the relatively high survival of *T. sebifera* plants in a long-term common garden experiment in Waimanolo (∼6 km from the Maunawilli garden, Siemann and Rogers 2003*b*) may be due to the benign conditions from aggressive management of background vegetation which changed the competitive environment (Joshi *et al.* 2014; Zheng *et al.* 2015*a*) or fine-scale temporal and/or spatial variation in the abiotic and/or biotic environment (Gabler and Siemann 2012). Likewise, we observed high rates of herbivory in the 17th year of that long-term experiment and in the first year of this study but the abundant herbivores were an Asian beetle (*Adoretus sinicus*, Scarabaeidae) and Caribbean slug, respectively, which may have very different interactions with plants at ecological and evolutionary time scales. Moreover, episodic outbreaks can have large effects on ecological communities (Carson and Root 2000) and evolution of plant traits (Bossdorf 2013; Uesugi and Kessler 2013) that may difficult to capture even in a three growing season experiment as we performed here when outbreaks are rare in time but very intense. Indeed, because plants may differ in their responses to abiotic and biotic environments over small differences in phenology, this can cause large variation among years in recruitment success (Gabler and Siemann 2013). This has been suggested to make it difficult to disentangle the roles of plant traits and environmental variation (such as rare events), especially for long-lived invasive plants (Colautti *et al.* 2004). If, however, plants from invasive populations have acquired traits that make them more fit in their native range over broad temporal and spatial scales compared with those from native populations, this suggests some process other than simple directional selection may be responsible for this adaptation (Lee 2002; Bock *et al.* 2015).

Large differences among venues and gardens in plant survival and plant growth even with insect suppression suggest that some other biotic factors contribute to differences in plant performance (Table 5). For example, it has been suggested that native plants in general have more negative interactions with the soil biota compared to exotic plants (Klironomos 2002) which has been found in a some studies (Zuppinger-Dingley *et al.* 2011; Andonian *et al.* 2012; Yang *et al.* 2013; Gundale *et al.* 2014; Dostalek *et al.* 2016) but sometimes has not been observed (Chiuffo *et al.* 2015; Otfinowski *et al.* 2016). This is thought to be driven by two possible mechanisms: greater diversity and density of soil pathogens on natives, especially near conspecifics (Meisner *et al.* 2014; van der Putten 2014) and potentially more beneficial interactions with mycorrhizae for exotics (Richardson *et al.* 2000*a*) though functional group may be at least as important as whether a plant is exotic or native (Bunn *et al.* 2015). In fact, there is evidence from the literature that both negative and positive interactions with the soil biota could have contributed to the geographic venue patterns we observed here. In a greenhouse experiment with *T. sebifera* and congeneric pairs of US and China tree seedlings, Yang *et al.* (2103) found that *T. sebifera* plants had more beneficial interactions with the soil biota in its introduced range compared to its native range relative to these other species. Moreover, in a field experiment with plants in pots in replicate gardens in the native and introduced ranges, *T. sebifera* plants had greater frequency of mycorrhizal association in the introduced range and *T. sebifera* plants from populations in the introduced range had greater frequencies of association than populations from the native range (Yang *et al.* 2015). Because there was no manipulation of the soil biota in this study and it would have been very difficult to collect roots of *T. sebifera* plants in these experiments in dense background vegetation, we could not easily assess the contribution of the soil biota, such as mycorrhizae to the differences among venues that we observed even when aboveground insects were suppressed. Moreover, there are a number of herbivores that feed belowground on *T. sebifera* plants in the native range, including both generalists and specialists (Zhang *et al.* 2015), that could have contributed to differences in performance among venues even with insecticide sprays. Nonetheless, the strong venue effects together with the large variation among gardens within a venue suggest that abiotic and/or biotic factors (other than aboveground insect herbivores) have potentially important effects on plant performance and perhaps also on evolution of plant traits (Erfmeier 2013; Zheng *et al.* 2015*b*).

**Table 5.** Summary of results versus predictions along with effect sizes. # refers to the predictions at the end of the introduction.

#	Predictor and response variables	Prediction	Result	Effect size	Matches prediction?
1	Venue: damage in control (no spray) treatments	HI>CN>TX	HI>CN>TX	(year 1) 7.2: 6.1: 1	**Yes**
	Venue: survival in control (no spray) treatments	TX>CN>HI	TX>CN>HI	1: 2.5: 2.9	**Yes**
	Venue: height in control treatments	TX>CN>HI	TX>CN>HI	(year 3) 1: 0.61: N/A	**Yes**
	Venue: mass in control treatments	TX>CN>HI	TX>CN>HI	3.2: 1: N/A	**Yes**
2	Origin: survival in Texas in control treatments	US>CN	US∼CN		No
	Origin: height in Texas in control treatments	US>CN	US>CN	(year 3) 1.2: 1	**Yes**
	Origin: mass in Texas in control treatments	US>CN	US>CN	1.3: 1	**Yes**
	Origin: survival in China in control treatments	CN>US	CN∼US		No
	Origin: height in China in control treatments	CN>US	US>CN	(year 3) 1.1: 1	No
	Origin: mass in China in control treatments	CN>US	US>CN	1.2: 1	No
3	Venue: damage in spray treatments	HI=CN=TX	HI>CN>TX	(year 1) 7.0: 4.2: 1	no
	Venue: survival response to insect suppression	HI>CN>TX	*variation among gardens within venue*		No
	Venue: height response to insect suppression	HI>CN>TX	HI∼CN∼TX		No
	Venue: mass response to insect suppression	HI>CN>TX	HI∼CN∼TX		No

Herbivore damage may not predict plant performance when there is variation in herbivore tolerance such as may be found between invasive and native plant populations (Stowe *et al.* 2000; Müller-Schärer *et al.* 2004; Wise and Abrahamson 2005). Such differences in herbivore tolerance could have contributed to our results. *Triadica sebifera* plants from US populations have been shown to have higher tolerance to herbivory from artificial defoliation (Rogers and Siemann 2004; Rogers and Siemann 2005), generalist herbivores (Zou *et al.* 2008; Carrillo *et al.* 2014) and specialist herbivores (Huang *et al.* 2010; Wang *et al.* 2011). In addition, experiments with artificial defoliation or generalist or specialist herbivores in cages have all found that US populations of *T. sebifera* plants outperform those from China even with higher levels of aboveground and belowground attack (Huang *et al.* 2012*b*; Carrillo and Siemann 2016) or repeated episodes of 100% defoliation (Wang *et al.* 2016). This type of result has been found for other invaders in which rapid growing invasive genotypes have higher tolerance to damage than ones from native populations (Meyer and Hull-Sanders 2008; Ridenour *et al.* 2008; Abhilasha and Joshi 2009). So, the greater performance of *T. sebifera* plants from US populations we observed here across a range of herbivore damage amounts is not surprising in the context of these studies but it is not clear why *T. sebifera* plants from native populations were able to outcompete ones from invasive populations in the earlier Hawaii common garden experiment (Siemann and Rogers 2003*b*).

Our results that showed large differences in survival and/or growth among gardens within a venue argue strongly for the importance of having replicate gardens within a venue, a practice that is still not the norm in such studies (but see Flory *et al.* 2011 for a great example of garden replication). At the extreme, if we had only had the Forestry garden in China and the Katy garden in Texas, we might have concluded that *T. sebifera* plants have higher performance in the native range which is the opposite of the result with replicate gardens. We have too few gardens to identify the factors that drove differences in survival and growth among gardens but our effective insect suppression treatments suggest that these factors must be something other than differences in aboveground herbivore attack. Indeed, from a scientific perspective scientists should include the maximum number of gardens in each range to be able to make strong inferences about differences among ranges and the number of gardens should be as high as possible given logistical constraints. However, the variation among gardens we observed does not preclude release from aboveground insect herbivory being an important selective pressure on plant traits, such as growth rate or competitive ability (Blossey and Nötzold 1995), but rather that other factors appear to be important in determining plant performance in particular conditions that vary within a range. In fact, if release from herbivores is consistent among sites and years, it may be important for evolution of plant traits while other factors that drive variation among sites and years may be important for ecological experiments but not a consistent selective force (Mauricio 2000; Roy and Kirchner 2000; Orians and Ward 2010).

There are a number of limitations to this study that could be addressed in future studies. First, the strength of tests of the EICA hypothesis are limited in that genetic differences do not imply selection for increased competitive ability in the non-native range. But, it has also been argued that inferences about determinants of invasion success are stronger when experiments include not only multiple ranges (Hierro *et al.* 2005), plants from replicate populations (Blair and Wolfe 2004) and experimental manipulation (Mitchell *et al.* 2006) as we have here but also multiple plant species (Agrawal *et al.* 2005; Liu and Stilling 2006) and consider a range of biotic interactions (van der Putten *et al.* 2004; Callaway and Maron 2006), ideally with factorial experimental manipulations (Mitchell *et al.* 2006; Orians and Ward 2010). The results of this study together with others with this focal invasive species point to simultaneous manipulation of soil organisms and herbivores as a particularly critical next step. However, the difficulties of conducting experiments that capture lifetime fitness of experimental plants suggests that the ability to test some dimensions of EICA hypothesis, such as those related to selection on plant traits, are limited by the long generation time of this focal species and perhaps could be addressed more productively with another focal species.

## Conclusions

The results of this study support a role for genetic differences between invasive and native populations of *T. sebifera* in its invasion success. In particular, more rapid aboveground growth rates appear to contribute to its success in both the invasive and native ranges. However, strong variation among sites indicates that *T. sebifera* plants from invasive populations only have a strong advantage in a subset of sites in China or Texas. The patterns for venues were consistent with the status of *T. sebifera* in that performance was high in Texas where it is invasive, intermediate in China, and low in Hawaii where it is introduced but not invasive. Together the results of this study suggest that differences in the traits of invasive plants consistent with release from natural enemies play a role in the greater competitive ability of plants from invasive populations but that invasion success also depends strongly on local environmental conditions.

## Sources of Funding

Our work was funded by the United States National Science Foundation (Award number DEB-0315796).

## Contributions by the Authors

W.E.R. and E.S. conceived the experiments. All authors designed and carried out the experiments, analyzed data, and participated in the writing of the manuscript.

## Conflicts of Interest Statement

None declared.

## Supplementary Material

Supplementary Data
