# Supplementary material for: An experimental test of the EICA hypothesis in multiple ranges: invasive populations outperform those from the native range independent of insect herbivore suppression
Source: AoB Plants. 2016 Dec 30;9(1):plw087. doi: 10.1093/aobpla/plw087 (PMC5499768; doi:10.1093/aobpla/plw087)
Supplement: Supplementary Data [file plw087_Supp.docx]

**Table S1.** AIC values for different models examining chewing damage (years 1, 2, and 3), height (years 1, 2 , and 3), survival time, mass, and percent leaves. O=Origin, S=Spray, V=Venue, G=garden. Bold values indicate the models selected.

|  |  |  |  |  |  |  |  |  |  |
| --- | --- | --- | --- | --- | --- | --- | --- | --- | --- |
| **Model parameters** | **D1** | **D2** | **D3** | **H1** | **H2** | **H3** | **Surv** | **mass** | **%leaf** |
| O | 4081 | 2727 | 1341 | 10081 | 7414 | 5696 | 12575 | 5091 | 2398 |
| S | 4078 | 2727 | 1337 | 10087 | 7417 | 5699 | 12578 | 5095 | 2387 |
| V | 3904 | 2727 | 1277 | 9761 | 7350 | 5669 | 12273 | 5066 | 2380 |
| O S | 4079 | 2724 | 1335 | 10074 | 7405 | 5685 | 12577 | 5082 | 2383 |
| V G(V) | 3617 | 2711 | 1265 | 9312 | 6821 | 5182 | 11789 | 4856 | 2371 |
| V O | 3906 | 2723 | 1274 | 9729 | 7337 | 5654 | 12275 | 5053 | 2377 |
| V S | 3901 | 2723 | 1274 | 9754 | 7341 | 5658 | 12275 | 5057 | 2366 |
| O S O*S | 4080 | 2720 | 1334 | 10066 | 7395 | 5673 | 12579 | 5072 | 2379 |
| V G(V) O | 3619 | 2706 | 1262 | 9267 | 6801 | 5162 | 11791 | 4841 | 2368 |
| V G(V) S | 3613 | 2708 | 1261 | 9305 | 6811 | 5172 | 11791 | 4847 | 2356 |
| V O S | 3903 | 2720 | 1271 | 9722 | 7328 | 5644 | 12277 | 5044 | 2362 |
| V O V*O | 3906 | 2716 | 1274 | 9712 | 7314 | 5642 | 12279 | 5041 | 2373 |
| V S V*S | 3893 | 2718 | 1268 | 9738 | 7319 | 5645 | 12276 | 5046 | 2362 |
| V G(V) O S | 3615 | 2704 | 1258 | 9260 | 6792 | 5152 | 11793 | 4832 | 2352 |
| V G(V) O V*O | 3619 | 2700 | 1262 | 9251 | 6780 | 5151 | 11795 | 4829 | 2364 |
| V G(V) S V*S | 3602 | 2702 | 1255 | 9291 | 6791 | 5160 | **11789** | 4837 | 2352 |
| V O S O*S | 3905 | 2716 | 1270 | 9714 | 7318 | 5631 | 12278 | 5033 | 2358 |
| V O S V*O | 3904 | 2713 | 1271 | 9705 | 7305 | 5631 | 12281 | 5032 | 2358 |
| V O S V*S | 3895 | 2714 | 1265 | 9706 | 7306 | 5631 | 12278 | 5033 | 2358 |
| V G(V) O S O*S | 3617 | 2700 | 1257 | 9253 | 6783 | 5141 | 11794 | 4822 | 2348 |
| V G(V) O S V*O | 3616 | 2697 | 1258 | 9244 | 6771 | 5141 | 11797 | 4821 | 2349 |
| V G(V) O S V*S | 3604 | 2698 | 1252 | 9246 | 6772 | 5140 | 11791 | 4822 | 2348 |
| V G(V) O V*O G*O(V) | 3619 | 2689 | 1260 | 9206 | 6742 | 5129 | 11800 | **4805** | 2358 |
| V G(V) S V*S G*S(V) | **3601** | **2692** | **1255** | 9248 | 6757 | 5148 | 11794 | 4827 | 2349 |
| V O S O*S V*O | 3905 | 2709 | 1270 | 9698 | 7295 | 5619 | 12282 | 5022 | 2355 |
| V O S V*S O*S | 3896 | 2710 | 1264 | 9699 | 7296 | 5618 | 12280 | 5023 | 2354 |
| V O S V*S O*S | 3896 | 2710 | 1264 | 9699 | 7296 | 5618 | 12280 | 5023 | 2354 |
| V O S V*S V*O | 3896 | 2707 | 1265 | 9690 | 7283 | 5618 | 12282 | 5021 | 2355 |
| V G(V) O S O*S V*O | 3617 | 2693 | 1257 | 9237 | 6762 | 5130 | 11798 | 4811 | 2345 |
| V G(V) O S V*O G*O(V) | 3615 | 2686 | 1256 | 9199 | 6732 | 5120 | 11802 | 4797 | **2343** |
| V G(V) O S V*S G*S(V) | 3604 | 2688 | 1252 | 9203 | 6738 | 5128 | 11796 | 4812 | 2345 |
| V G(V) O S V*S O*S | 3606 | 2694 | 1252 | 9239 | 6762 | 5129 | 11791 | 4812 | 2344 |
| V G(V) O S V*S O*S | 3606 | 2694 | 1252 | 9239 | 6762 | 5129 | 11791 | 4812 | 2344 |
| V G(V) O S V*S V*O | 3605 | 2692 | 1252 | 9230 | 6751 | 5129 | 11795 | 4811 | 2345 |
| V O S V*S O*S V*O | 3897 | 2703 | 1264 | 9682 | 7273 | 5606 | 12283 | 5011 | 2351 |
| V O S V*S O*S V*O | 3897 | 2703 | 1264 | 9682 | 7273 | 5606 | 12283 | 5011 | 2351 |
| V G(V) O S O*S V*O G*O(V) | 3617 | 2682 | 1255 | 9192 | 6723 | 5108 | 11802 | 4787 | 2339 |
| V G(V) O S V*S G*S(V) O*S | 3605 | 2684 | 1251 | 9196 | 6729 | 5117 | 11796 | 4802 | 2341 |
| V G(V) O S V*S O*S V*O | 3606 | 2687 | 1251 | 9223 | 6742 | 5118 | 11795 | 4801 | 2341 |
| V G(V) O S V*S O*S V*O | 3606 | 2687 | 1251 | 9223 | 6742 | 5118 | 11795 | 4801 | 2341 |
| V O S V*S O*S V*O V*O*S | 3895 | 2695 | 1261 | 9664 | 7249 | 5592 | 12287 | 4999 | 2345 |
| V G(V) O S V*S G*S(V) O*S V*O G*O(V) | 3605 | 2666 | 1249 | 9135 | 6670 | 5085 | 11805 | 4767 | 2332 |
| V G(V) O S V*S G*S(V) O*S V*O G*O(V) | 3605 | 2666 | 1249 | **9135** | **6670** | **5085** | 11805 | 4767 | 2332 |
| V G(V) O S V*S G*S(V) O*S V*O G*O(V) V*O*S | 3604 | 2657 | 1246 | 9117 | 6647 | 5070 | 11808 | 4755 | 2326 |
| V G(V) O S V*S G*S(V) V*O G*O(V) | 3604 | 2670 | 1250 | 9143 | 6679 | 5096 | 11804 | 4777 | 2336 |
| V G(V) O S V*S O*S V*O V*O*S | 3605 | 2679 | 1248 | 9205 | 6719 | 5103 | 11799 | 4789 | 2335 |
| V G(V) O S V*S G*S(V) O*S V*O G*O(V) V*O*S G*O*S(V) | 3598 | 2644 | 1243 | 9078 | 6612 | 5057 | 11816 | 4743 | 2322 |
|  |  |  |  |  |  |  |  |  |  |

**Fig. S1** A. The locations of source populations (green trees) in the B. native and C. invasive range and gardens in the D. native and non-native ranges (E. Hawaii, F. Texas) used in the experiment. <https://www.google.com/maps> [accessed August 3, 2016).
